# Research Progress on Testicular Cancer in Giant Pandas: A Case-Oriented Review

**DOI:** 10.3390/vetsci12060544

**Published:** 2025-06-03

**Authors:** Chengdong Wang, Chengyao Li, Linhua Deng, Meg Sutherland Smith, Rongping Wei, Li Luo, Shanshan Ling, Lixian Liu, Benjamin Nevitt, Bo Luo, Wenjing Li, Caiwu Li, Haidi Yang, Ming Wei, Ti Li, Kai Wu, Yuanyuan Qu, Desheng Li

**Affiliations:** 1 China Conservation and Research Center for the Giant Panda, Key Laboratory of SFGA on the Giant Panda, Chengdu 610081, China; 2 San Diego Zoo Wildlife Alliance, San Diego Zoo Global, 2920 Zoo Drive, San Diego, CA 92101, USA; 3 Chengdu Research Base of Giant Panda Breeding, Chengdu 610081, China; 4 Shijiazhuang Zoo, Shijiazhuang 050200, China; 5 Sichuan Wolong National Nature Reserve Administration, Aba Tibetan and Qiang Autonomous Prefecture, Wenchuan 623006, China

**Keywords:** giant panda, testicular tumor, clinical examination, castration, familial inheritance

## Abstract

The giant panda (*Ailuropoda melanoleuca*), as an endemic and endangered species in China, has attracted much attention regarding its health issues. In recent years, certain diseases that affect giant pandas have gradually become important factors impacting their survival and breeding, including tumor diseases. Research on testicular cancer in giant pandas, in particular, has attracted the attention of scientific researchers because it poses a threat to individual reproductive function and population reproduction. This review systematically reviews the research progress of testicular cancer in giant pandas in recent years. Firstly, starting from case reports, the current research status of clinical manifestations, diagnostic methods and molecular mechanisms of testicular cancer in giant pandas was analyzed. Secondly, the commonly used treatment methods at present were discussed, including surgical resection, radiotherapy and chemotherapy, etc., and the advantages and disadvantages of these methods in practical applications were summarized. Furthermore, this article also conducts an in-depth exploration of the pathogenesis of testicular cancer in giant pandas, involving possible triggers such as genetic factors, environmental factors, and endocrine disorders. Based on the existing research results, this paper proposes a few future research directions, which will provide important support for the health management and population protection of giant pandas. Overall, although certain progress has been made in the research of testicular cancer in giant pandas, it still needs to be further deepened in order to provide a more powerful scientific basis for the healthcare and breeding of the endangered giant panda species.

## Simple Summary

The giant panda, an endemic and endangered Chinese species, has health issues drawing much attention, with tumor diseases including testicular cancer being a concern as it threatens reproduction. This review analyzes the research progress on giant panda testicular cancer. It starts with case reports to study clinical manifestations, diagnostic methods and molecular mechanisms. It also discusses common treatments like surgery, radiotherapy, and chemotherapy, summarizing their pros and cons. The pathogenesis, involving genetic, environmental, and endocrine factors, is explored. Future research directions are proposed to support panda health management and protection. More in-depth research is still needed for panda healthcare and breeding.

## 1. Introduction

The giant panda, as China’s national treasure and a key species for conservation efforts, has seen population growth due to government protection measures [1,2]. As conservation efforts move into a new phase, shifting from a purely quantitative focus to a more holistic focus on individual health will help ensure the long-term prosperity and development of this precious species. As the captive population expanded, so did the number of oncological diseases such as liver cancer, osteosarcoma, skin cancer, ovarian cancer, and testicular cancer. According to clinical statistics, the highest incidence of giant panda tumors is testicular tumors with a total of eight cases.

Testicular tumors can be divided into germ cell tumors, non-germ cell tumors, and secondary testicular tumors [3]. In humans, 95% of testicular tumors are testicular germ cell tumors, which are mainly divided into two categories: seminoma and non-seminoma, of which seminoma accounts for 40–45% [4,5]. In the veterinary field, especially in domestic and companion animals (dogs), the incidence of testicular cancer is relatively high; studies have reported a significant increase in the prevalence of dogs that have not undergone castration surgery, and some breeds are more susceptible due to genetic factors [6]. In recent years, with the rapid development of genomics, proteomics, and imaging technology, remarkable progress has been made in the study of animal testicular cancer, but its pathogenesis still needs to be further explored [7,8]. The typical manifestations of testicular tumors in giant pandas are abnormal testicular swelling, increased hardness, local pain, and local fever in a short period of time during the non-breeding period [9]. The diagnosis can be confirmed by clinical palpation, ultrasound, computed tomography (CT) examination, cytology of aspirated samples, etc. Generally, this condition is cured after castration surgery, and no cancer cell metastasis is found before and after surgery.

In this paper, the general situation, epidemiology, potential pathogenesis, clinical diagnosis, treatment, protection measures, and health management of testicular tumor of the giant panda were reviewed.

## 2. Overview of Testicular Cancer in Giant Pandas

All giant panda breeding institutions at home and abroad were investigated, and cases of giant pandas breeding in recent years were screened through historical reviews, investigations, and visits. Up to now, a total of eight cases of orchiectomy in giant pandas have been reported. All cases of testicular tissue resection were confirmed as seminoma by pathological section, and relevant information is shown in Table 1. The average age at diagnosis/surgery was 19.4 years old, with the youngest being 12.5 years old and the oldest at onset being 26.5 years old. The oldest age for recurrence was 31 years old. Surgical procedures included bilateral resections in five cases, left-side resections in two cases, and a single right-side resection.

According to relevant statistics, six of the eight giant pandas with testicular tumors successfully collected sperm. Among them, sperm quality was poor in one case, fine in three cases, and medium in two cases. Four giant pandas (Xing-Xing, Gao-Gao, Mei-Sheng, and Xi-Meng) were able to mate naturally before they became ill. Apart from Mei-Sheng, the other three giant pandas have had offspring, but Xing-Xing’s offspring died and did not grow up.

## 3. Epidemiology, Genetics, and Potential Pathogenesis Analysis

**Figure 1:**
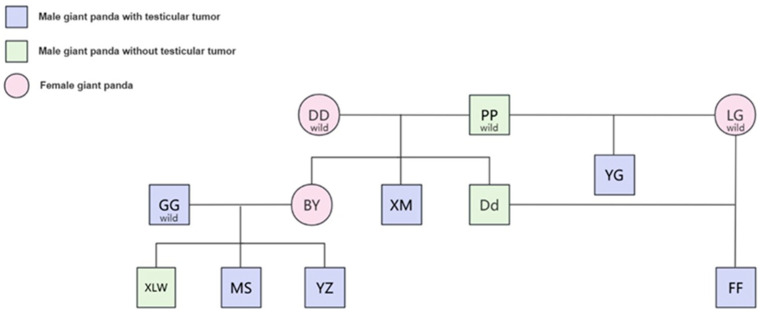
Family atlas of giant panda with testicular tumor. DD, PP, LG, GG, BY, XM, Dd, YG, XLW, MS, YZ, and FF are the abbreviations of the giant pandas Dong-Dong, Pan-Pan, Long-Gu, Long-Gu, Gao-Gao, Bai-Yun, Xi-Meng, Da-Di, Yang-Guang, Xiao-Liwu, Mei-Sheng, Yun-Zi, and Fu-Fu, respectively.

Testicular cancer is one of the most common tumors in males. In humans, testicular cancer is the most common solid cancer in men aged 25 to 34 and is usually curable, especially seminoma, which accounts for about 1% of male tumors [12]. In animals, mainly in dogs, testicular cancer is one of the most common tumors in elderly uncastrated dogs, but its overall incidence is not high, mainly because most dogs have undergone past potential surgery [13]. Up to now, there have been a total of eight recorded cases of testicular cancer in giant pandas, accounting for about 2% of male pandas in captivity, and the incidence of human incidence is relatively close; the age of onset is mostly the middle-aged and elderly, and the age of onset is equivalent to 58 years in human age. Studies have shown that the risk of testicular cancer is largely due to a man’s genotype, and that genetic inheritance is more important for testicular cancer than for other types of cancer. In humans, having a brother with testicular cancer increases one’s own risk 8–10-fold; if a father has testicular cancer, his son’s risk is increased 4–6-fold [14]. Genetic analysis revealed that giant pandas with testicular tumors were primarily concentrated in the offspring of wild pandas Pan-Pan and Gao-Gao from Baoxing, Sichuan. Notably, Xi-Meng, a son of Pan-Pan and Dong-Dong, had the condition, while their other son, Da-Di, was healthy. Interestingly, Fu-Fu, the son of Da-Di (whose mother, Long-Gu, is also from Baoxing), suffered from testicular tumors. Additionally, Yang-Guang, another offspring of Pan-Pan and Long-Gu, was diagnosed with the condition. In the San Diego Zoo, Bai-Yun, daughter of Pan-Pan and Dong-Dong, had five cubs with Gao-Gao. Unfortunately, Gao-Gao and his two sons, Mei-Sheng and Yun-Zi, developed testicular tumors and underwent surgery. Only their son, Xiao-Liwu, survived (Figure 1). This result shows that the risk of testicular cancer in giant pandas is greatly influenced by genetic factors, and it seems that female genes in addition to male genes also play a crucial role in the disease of testicular cancer. Pan-Pan is not a testicular tumor patient, has impregnated several female pandas, and has numerous offspring. However, cases have only appeared in the descendants of Dong-Dong or Long-Gu. Currently, in humans, there have also been reports that testicular cancer has suggested a recessive mode of inheritance, with a penetrance among homozygotes of 43%. Both maternal CYP1B1 variants and index subjects’ CYP3A4 and CYP3A5 variants were found to be associated with an increased risk of testicular cancer [15]. However, given the small number of giant pandas, it seems that this conclusion can only be defined as extrapolation, because there are no more accurate statistics on the incidence and probability of tumors.

Furthermore, in our current review and case study, we have not conducted a direct investigation into inbred lines. The main reason lies in the fact that it is quite difficult to obtain comprehensive pedigree information for accurately calculating inbred lines. Although there is a certain accumulation of genealogical records of giant pandas, some early records have information deficiencies or inaccuracies. Especially for some individuals that were rescued from the wild and kept in captivity for breeding, it is difficult to trace their parental information completely. However, we recognize that inbred lines may be of great significance for understanding the pathogenesis of testicular cancer. In the subsequent research, we will comprehensively sort out and improve the genealogy database of giant pandas. Meanwhile, by using advanced genotyping techniques, such as genotyping based on single nucleotide polymorphisms (SNPS), the genetic relationship between individuals can be inferred more accurately from a genetic perspective, and then the number of inbred lines can be estimated to deeply explore its association with the occurrence of testicular cancer.

The current research on panda testicular cancer focuses on clinical diagnosis, treatment, and diagnostic markers. Chen et al. conducted single cell sequencing of the testicular tissue removed from Fu-Fu, including normal tissue on the outside, tumor tissue in the middle, and necrotic tissue in the center. They found that germ cell markers were expressed in almost all tumor cells, and that the tumor cells appeared to be the same subtype of seminoma cells. In addition, four clusters with unique gene expressions, including early apoptotic cells (EAC), inactivated cells (IC), active cell subcluster 1 (AC-1), and active cell subcluster 2 (AC-2), were identified. At the same time, studies have also proposed that CD117 and CD30 can be used as reliable markers for future pathological diagnosis [11]. In the study by Zhu et al., the two items of blood small RNA and blood exosomal small RNA were compared between pandas with testicular cancer and normal pandas, and the results showed that the level of miR-331-3p in the blood or blood exosomes of pandas with testicular tumor was increased, and this result was also verified by qPCR. This suggests that miR-331-3p may be used as a diagnostic marker for testicular tumors of giant pandas [10]. However, the pathogenesis of testicular cancer in giant pandas has not been reported. We reviewed the literature on testicular cancer in humans and dogs and summarized its potential pathogenesis, hoping to provide a reference for future research on giant pandas. In human studies, genome-wide association studies have identified 78 TGCT susceptibility sites, many of which are in non-coding regions, suggesting that non-coding RNAs are involved in TGCT pathogenesis. Mrinal et al. suggested that miRNAs may act as oncogenes or tumor suppressors of testicular cancer by regulating targets involved in cell proliferation, apoptosis, and metastasis [16]. In dogs, cryptorchidism (the failure of the testicle to descend into the scrotum) is considered an important risk factor for testicular tumor development [17]. The incidence of testicular tumors in dogs with cryptorchidism is 13–14-fold higher than that in normal dogs. In addition, the higher ambient temperature of cryptorchidism has also been suggested as a possible cause.

## 4. Diagnosis of Testicular Tumor in Giant Pandas

**Figure 2:**
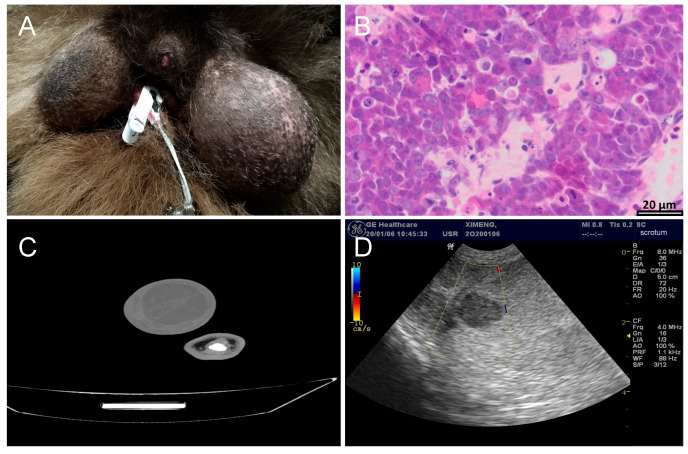
Diagnosis results of giant pandas with testicular tumors. (**A**) Enlargement of the left testicle of giant panda Xi-Meng. (**B**) HE staining sections of testicular histopathology of giant pandas with testicular tumors. (200×). (**C**) CT scans of giant panda Ya-Xiang. (**D**) Ultrasound examination results of the left testicle of Xi-Meng.

The main clinical symptoms were an abnormal enlargement of testis 2–3-fold or more in a short period of time during the non-breeding period, and the size of the left and right sides was significant compared to unilateral cases (Figure 2A). Male giant pandas with testicular cancer show a weak lateral walking posture during the non-breeding period, an abnormal urination posture similar to that of females in the squatting position, and frequent friction or marking of the perineum. At the same time, sparse scrotal hair, swollen scrotal skin, and local heat can be observed. During palpation, the touch is sensitive, and the texture is uneven. The lesion area has a relatively solid texture. However, there were no abnormal weight loss or abnormal manifestations in terms of mental state and appetite.

In addition to clinical symptom examination, laboratory examination is also one of the reference indicators for the diagnosis of testicular cancer. Mainly for serum β-HCG, AFP, and LDH detection, these serum tumor markers are important for treatment, follow-up, and prognosis. In the report of Zhang et al., the serum levels of AFP and LDH in giant panda Mei-Sheng were significantly increased [9]. This also coincides with the detection of tumor markers for human testicular cancer.

Histopathological examination is the “gold standard” for the diagnosis of most diseases, especially tumor diseases [18]. At present, all testicular tumor cases in giant pandas are pathologically diagnosed as “seminoma”. HE staining of the excised testicular tissue showed that the common features of multiple cases were the loss of basic testicular structure, the replacement of the original hollow tubule spermatogenic tubule by parenchymal cells, and the absence of a curved tubule structure. Parenchymal cells are distributed in the form of clumps or sheets, with large volume, round or polygon shape, clear cell boundaries, clear staining of nuclei, blister-like shape, obvious nucleolus, abundant cytoplasm, and visible mitotic images. These features are also mostly malignant transformation phenomena (Figure 2B).

Imaging is a central tool in the diagnosis and staging of testicular cancer. The AUA’s latest guidelines recommend that in patients with newly diagnosed germ cell tumors (GCT), physicians should prioritize enhanced CT scans of the abdomen and pelvis [19,20]. This is because posterior peritoneal lymph nodes are the most common site of metastatic testicular cancer. The sensitivity of CT is 67%, and the specificity is 95% [1], which has important reference value for tumor staging and treatment decision. In addition, CT can also assess the extent of tumor spread and provide a basis for subsequent treatment options.

The CT scan results of giant pandas with testicular tumors show that testicular tumors were mostly circular, with mixed low density on plain scan, and the solid part was mainly uniform enhancement. CT images showed that the contours of the diseased testis were 2–3 times larger than the normal testis, and the contours were convex and convex. The texture is uneven, shows multiple dark areas and shadows, has cystic boundaries, and is slightly denser than normal tissue. Cystic and calcification foci were seen inside the tumor (Figure 2C). Abdominal CT provides three-dimensional tumor size and tissue relationship details, distinguishing solids from cystic masses with 90–100% accuracy and identifying central necrotic liquefaction, thereby enhancing testicular tumor diagnosis accuracy and serving as a valuable diagnostic tool [21].

In addition to CT scans, the imaging assistance methods for testicular tumors also include ultrasound examinations. Color Doppler ultrasonography is a key imaging method for diagnosing scrotal and testicular diseases due to its simplicity and non-invasiveness [22]. It achieves an 85% detection rate and 44.4% specificity for testicular tumors [23], aiding in distinguishing between benign and malignant tumors, torsion, orchitis, injury severity, and hematoma presence through blood flow analysis [24]. Ultrasonography can accurately distinguish the size, shape, and mass of the testis, and can also distinguish whether the swollen testis is caused by inflammation, tissue edema, or a tumor, and can also detect whether there is a retroperitoneal metastatic tumor. Ultrasonographic features of testicular tumors include enlargement, non-uniform internal echogenicity, round shape with clear boundaries, and potential multiple locations (Figure 2D). High-resolution ultrasound can detect tumors as small as 3.0 mm, enabling accurate diagnosis. However, in cases without characteristic tumor morphology and multi-vessel abnormalities, ultrasonography may struggle to differentiate between inflammation and tumors, necessitating additional clinical data for objective assessment [21].

## 5. Treatment

The treatment method for this disease involves castration to remove the diseased testicles. Currently, two common surgical approaches have emerged: anterior closed scrotal castration and closed scrotal castration. Among them, the traditional surgery adopts anterior closed scrotal castration. The skin is slit in the middle of the head/tail direction, and the subcutaneous tissue is cut at the top of each testicle to complete the castration and suture. This method requires a focus on checking the firmness of the spermatic cord ligation. The vas deferens should be ligated separately to prevent bleeding. Depending on the situation, a drainage strip can be inserted to prevent hematoma exudation. At the same time, antibiotic treatment for 5 to 7 days should be used for infection control. In 2024, the surgery for Xi-Meng adopted a closed scrotal castration, while also removing part of the scrotal tissue. This surgery exposes the testicles by cutting open the skin and subcutaneous tissue. After separating the epididymal caudal ligament, the testicular sheath near the epididymis is clamped with a hemostatic forceps to identify and ligate the vas deferens, blood vessels, and spermatic cord. This improved surgery significantly reduces the risks of scrotal swelling and hemorrhagic secretions, while reducing suture materials and anesthesia time, and largely lowering the surgical cost. This method has gradually become the preferred option for the castration of animals such as dogs and cats and is particularly suitable for the postoperative care of giant pandas. All the giant pandas that underwent surgery were systematically followed up and observed. The data showed that they all recovered within two weeks after the operation, and no secondary infections occurred. the giant panda Xi-Meng, in particular, who was treated with the new surgical method in 2024, successfully recovered within a week of the operation, which also demonstrated the superiority of the new surgical method. During the long-term monitoring, only tumor features were found in the right testicle of Xi-Meng four years after the first left testicle resection. No recurrence was observed in the remaining individuals with unilateral resection, and the retained unilateral testicle still had the function of normal semen collection. To ensure postoperative health management, this review emphasizes the establishment of a standardized follow-up care system, including quarterly physical examinations, monitoring weight changes, and the remaining testicular status. Blood biochemical tests are conducted every six months to evaluate the levels of tumor markers. The annual comprehensive anesthesia examination is combined with CT scans to monitor potential cancer metastasis. Meanwhile, it is suggested that veterinarians and keepers strengthen postoperative medical training for giant pandas to ensure that they can adapt to the requirements of routine anesthesia-free examinations and specialized treatments, which is of great significance for long-term health management.

## 6. Outlook

**Figure 3:**
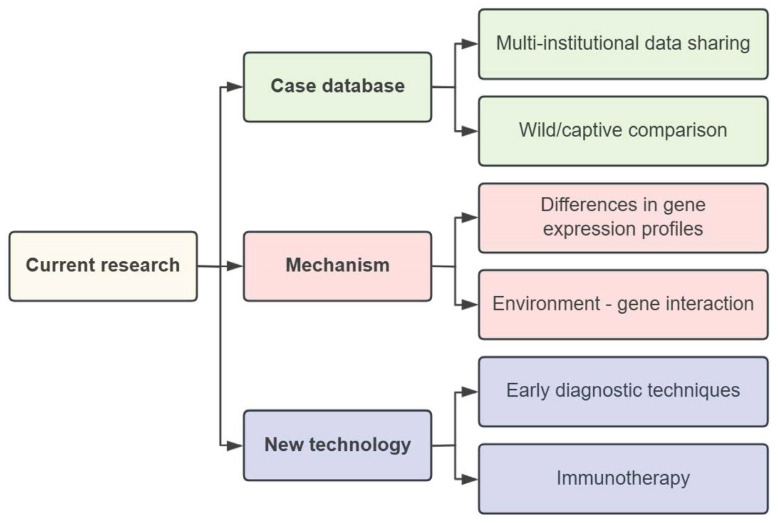
Overview diagram of subsequent research.

Building on the existing research, future studies on special cancers in giant pandas will primarily focus on three directions. Firstly, efforts will be dedicated to establishing a comprehensive case database. This involves creating a multi-institutional data-sharing platform and conducting comparative analyses of cases between wild and captive giant pandas, thereby enriching epidemiological research. Secondly, in terms of cross-species mechanisms, researchers will analyze differences in gene expression profiles and develop environment–gene interaction models. This includes mining genomic data to identify genetic and epigenetic markers associated with testicular cancer, comparing mutation profiles with those of other species, and studying epigenetic changes to understand the molecular mechanisms of tumorigenesis. It will also facilitate the screening of potential targeted drugs by focusing on key signaling pathways such as PI3K/AKT/mTOR and WNT/β-catenin. Finally, in the field of diagnostic and treatment technology innovation, research will focus on developing non-invasive screening techniques and optimizing immunotherapy regimens—for example, further screening tumor-associated antigens or microRNAs (such as exploring beyond miRNA-331-5p for early diagnosis), and tailoring CAR-T cell therapies or checkpoint inhibitors (such as PD-1/PD-L1 inhibitors) to the molecular characteristics of giant panda testicular cancer (Figure 3). In the future, the health monitoring of captive giant pandas should be strengthened and cross-species and cross-disciplinary cooperative research should be simultaneously carried out to provide a scientific basis for the prevention and treatment of this rare disease.

## 7. Conclusions

This review integrates the only eight publicly reported cases of testicular cancer in giant pandas globally, summarizing the disease from aspects such as etiology, diagnosis, and treatment methods. It is found that the disease primarily affects elderly male giant pandas, with seminoma being the common pathological type. However, the extremely small sample size and fragmented data severely restrict accurate judgments on disease incidence, risk factors, and prognosis. In the future, it will be necessary to establish standardized health monitoring systems, enhance cross-institutional data sharing and biobank construction, explore early diagnostic markers through molecular biology approaches, and optimize treatment protocols suitable for giant pandas to advance research in this field and contribute to the health protection of this endangered species.

## Figures and Tables

**Table 1:** Basic information of confirmed giant pandas.

Name (ID)	Birthplace	Age of Operation (Y/O.)	Ages (Y/O.)	Single/Bilateral	Semen Quality
Xing-Xing (112)	Baoxing (wild)	26.5	Post-surgical survival for 2 years (Pass away)	Right/Bilateral resection	No data
Gao-Gao (415) [10]	Baoxing (wild)	21.7	32.7	Right	Medium
Mei-Sheng (563) [9]	San Diego Zoo	14.8	21.7	Bilateral	Good
Yang-Guan (564) [10]	Wolong Base	14.5	21.7	Bilateral	Good
Xi-Meng (399) [10]	Wolong Base	26.3/31	31.6	Left/Right	Medium
Ya-Xiang (529) [10]	Chengdu Base	18.8	23.7	Right	Poor
Fu-Fu (532) [11]	Wolong Base	17.5	23.7	Left	Good
Yun-zi (749)	San Diego Zoo	12.5	15.8	Bilateral	No data
